# Supplementary material for: From sequence to enzyme mechanism using multi-label machine learning
Source: BMC Bioinformatics. 2014 May 19;15:150. doi: 10.1186/1471-2105-15-150 (PMC4229970; doi:10.1186/1471-2105-15-150)
Supplement: Additional file 2 — Java code of ml2db. Additional file ml2db_code.tar.gz contains the Java source code to run the multi-label machine learning experiments and save the results to database. The code’s Javadoc is included. [file 1471-2105-15-150-S2.zip › additional file 2/ml2db/utils/doc/index-files/index-23.html]

W-Index


JavaScript is disabled on your browser.


- Overview
- Package
- Class
- Use
- Tree
- Deprecated
- Index
- Help

- Prev Letter
- Next Letter

- Frames
- No Frames

- All Classes

A B C D E F G H I J K L M N O P Q R S T U V W X Y 


## W

WebUtils - Class in uk.ac.standrews.utils.main.webutils
:   Web utilities.

WebUtils() - Constructor for class uk.ac.standrews.utils.main.webutils.WebUtils


WebUtilsTest - Class in uk.ac.standrews.utils.test
:   Class

WebUtilsTest() - Constructor for class uk.ac.standrews.utils.test.WebUtilsTest


writeFileToTable(String, String, String, int, String) - Method in class uk.ac.standrews.utils.main.database.TableWriter
:   Writes a data file to table using LOAD DATA LOCAL INFILE return true if
    the statement was executed

writeRowToTable(TableRow) - Method in class uk.ac.standrews.utils.main.database.TableWriter
:   Insert the row values into the appropriate database table

writeToFile(String, String) - Static method in class uk.ac.standrews.utils.main.FileUtils

A B C D E F G H I J K L M N O P Q R S T U V W X Y

- Overview
- Package
- Class
- Use
- Tree
- Deprecated
- Index
- Help

- Prev Letter
- Next Letter

- Frames
- No Frames

- All Classes
